# Unusual Synchronous Methimazole-Induced Agranulocytosis and Severe Hepatotoxicity in Patient with Hyperthyroidism: A Case Report and Review of the Literature

**DOI:** 10.1155/2015/934726

**Published:** 2015-04-28

**Authors:** Jun Yang, Jun Zhang, Qin Xu, Guo-ping Sheng, Wan-wen Weng, Meng-jie Dong

**Affiliations:** ^1^The Department of Nuclear Medicine, the First Affiliated Hospital, College of Medicine, Zhejiang University, Hangzhou 310003, China; ^2^Collaborative Innovation Center for Diagnosis and Treatment of Infectious Disease, the First Affiliated Hospital, College of Medicine, Zhejiang University, Hangzhou 310003, China

## Abstract

*Context*. To report a patient with hyperthyroidism who developed concurrent occurrence of agranulocytosis and severe hepatotoxicity after taking methimazole (MMI). *Case*. A 51-year-old Chinese male was diagnosed as hyperthyroidism with normal white blood count and liver function. After 4 weeks' treatment with MMI 20 mg/d, it developed to agranulocytosis and severe cholestatic hepatotoxicity. The patient's symptoms and laboratory abnormalities disappeared after the withdrawal of MMI; his white blood count and liver function recover to normal in 2 weeks and 5 weeks, respectively. 296 MBq dose of ^131^I was given to the patient 3 weeks after the withdrawal of MMI and his thyroid function was back to normal in 6 months. As we know through literature review, only 5 previous cases reported the synchronous ATD-induced agranulocytosis and severe hepatotoxicity in patients with hyperthyroidism. *Methods*. Review of the patient's clinical course. Literature review of cases of hyperthyroidism with agranulocytosis and severe hepatotoxicity demonstrated that these complications occurred after taking antithyroid drug (ATD). *Conclusions*. Patient with hyperthyroidism can have synchronous ATD-induced agranulocytosis and severe hepatotoxicity. This case is extremely rare, but the adverse effects with ATDs is clinically significant. The clinicians need to be careful about this and monitor biochemical of patients who take ATDs.

## 1. Background

Antithyroid drugs (ATDs) have been cornerstones in the management of hyperthyroidism for six decades. ATDs, including methimazole (MMI), propylthiouracil (PTU), and carbimazole (CBZ), are widely used in different regions by physicians. ATDs are associated with a variety of side effects, like as skin rash, low-grade liver dysfunction, granulocytopenia, and arthralgia. Among these side effects, agranulocytosis, myeloperoxidase antineutrophil cytoplasmic antibody-related vasculitis, and severe hepatotoxicity are rare but serious and have potential lethal complications [1]. Agranulocytosis, defined as agranulocyte count below 500/L, is occurring in 0.1–0.5% of hyperthyroidism patients who receive ATD therapy and usually abruptly within the first 3 months and in the older patients who may be receiving high dose of ATD [2–7]. ATD-induced severe hepatotoxicity after patients take ATD is defined in the guidelines proposed by Tajiri and Shimizu [8]. Severe hepatotoxicity commonly occurs suddenly in the early stages of ATD treatment according to reviews [9–11]. The synchronous ATD-induced agranulocytosis and severe hepatotoxicity are extremely rare and only have been reported in a very few cases [12–16]. Here, we share our experience of managing a case of MMI-induced agranulocytosis and severe hepatotoxicity and review the previous reported cases on this unusual adverse effect of ATD therapy.

## 2. Case Presentation

A 51-year-old Chinese man came to our hospital complaining of weight loss of 4 kg, finger tremors, sweating, and palpitations for 1 month in May 2013. Physical examination showed that his thyroid gland was slightly enlarged and tender. No other clinically relevant physical findings were recorded. His thyroid function was presented as increased free thyroxine (FT4), free triiodothyronine (FT3) along with low thyroid-stimulating hormone (TSH). At that time, his blood count, liver function test, erythrocyte sedimentation rate, and C-reactive protein were within the normal range. A thyroid scan using ^99m^TcO_4_^−^ revealed diffuse increased uptake in the thyroid area and ultrasound showing increased color Doppler flow was in the symmetrically enlarged thyroid. He was diagnosed as having hyperthyroidism due to Graves' disease and started treatment with MMI (10 mg twice daily) and propranolol (10 mg thrice daily). Four weeks after taking these drugs, he had a sore throat with high fever (40.0°C), skin rash, cholestatic symptoms including dark urine, pruritus, and jaundice. He went to the hospital and got laboratory analyses. The results showed total WBCs 1.1 × 10^9^/L with no neutrophils, total serum bilirubin (TB) 385 *μ*mol/L, conjugated bilirubin (DB) 299 *μ*mol/L, alanine aminotransferase (ALT) 89 U/L, aspartate aminotransferase (AST) 30 U/L, prothrombin time (PT) 17 s, FT4 37.1 ng/dL, FT3 5.61 pg/mL, and TSH 0.001 *μ*lU/mL. He was recommended to stop MMI immediately. Further examination results were as follows: his electrocardiogram and ultrasonic cardiogram were normal; abdominal ultrasonograph showed no evidence of biliary dilatation or gallbladder disease; chest X-ray showed no abnormalities. He was negative for antinuclear antibody, anti-smooth-muscle antibody, antineutrophil cytoplasm antibodies, and hepatitis A, B, C, and E. He has no history of alcohol addiction, smoking, additional drug, blood transfusion, or recent overseas travel. So we considered this to be a diagnosis of MMI-induced severe cholestatic hepatotoxicity with agranulocytosis. Initial management included cessation of MMI, empiric antibiotic treatment (IV Sulperazone, oral levofloxacin) and hepatoprotective treatment (IV atomolam and glycyrrhizinate). By day 5, the patient was afebrile. At the 2nd week, his granulocytes count had risen to 3.1 × 10^9^/L, his liver function tests were all improving, and TB had fallen to 158 *μ*mol/L. Three weeks after MMI stopped, 296 MBq dose of ^131^I was given. Five weeks later, the values of liver function decreased to normal. His symptoms of hyperthyroidism were improved in the 3rd month after ^131^I treatment and his thyroid function remained at normal levels in the 6th month. During the course of the patient, changes in his thyroid function, liver function, and blood counts are shown in Table 1.

## 3. Methods

Pubmed and Embase search were performed between the year of 1950 and October 2014, in English language, limited to “human,” using the terms “antithyroid drug or methimazole or propylthiouracil orcarbimazole,” “agranulocytosis,” and “hepatotoxicity or liver dysfunction or hepatitis.” We collected the data from the review according to the following criteria. First, agranulocytosis was defined as agranulocyte count below 500/L after taking ATD. Second, the severe hepatotoxicity criteria in patients taking ATD according to the guidelines proposed by Tajiri and Shimizu are as follows [8]: ALT greater than 8x the upper limit of normal (ULN); ALT greater than 3x ULN in association with TB level greater than 2x ULN or prothrombin time international ratio (PT-INR) prolonged more than 1.5x; and symptoms of liver injury with TB greater than 3x ULN or a PT-INR prolonged more than 1.5x. Finally, we excluded the literature through which we could not obtain the accurate data of agranulocyte count and liver functions.

This case was approved by the institutional review board.

## 4. Results

There were 5 cases of synchronous ATD-induced agranulocytosis and severe hepatotoxicity. The clinical characteristics of the patients with synchronous ATD-induced agranulocytosis and severe hepatotoxicity are shown in Table 2. There are one male and five females, with a mean ± SD age of 46.2 ± 11.6 yr. Three patients were treated with CBZ, 2 patients were treated with PTU, and one patient was treated with MMI. The mean total duration of ATD treatment was 27.0 ± 9.4 days (mean ± SD). All patients were recommended to suspend ATD immediately when they were diagnosed as agranulocytosis and severe hepatotoxicity. Mean time of recovery of agranulocyte count and liver function is 18.0 ± 5.5 days and 54.8 ± 15.1 days in 4 patients (one was not reported). One patient died due to acute fulminating septicemia secondary to agranulocytosis. Four patients took radioiodine (one did not report) and 2 patients have euthyroid and 2 patients have subclinical hyperthyroidism in the follow-up.

## 5. Discussion

ATD-induced agranulocytosis and severe hepatotoxicity are the most serious adverse effects since ATD was introduced to manage the patient with hyperthyroidism for six decades. The prevalence of ATD-induced agranulocytosis is 0.1–0.5% [2–7]. Severe hepatotoxicity occurs in 0.1–0.2% and in the early treatment period [1, 5]. The concurrent occurrence of both agranulocytosis and severe hepatotoxicity induced by ATD therapy is extremely rare. To the best of our knowledge, it is the first case of MMI-induced agranulocytosis along with severe hepatotoxicity onset within 4 weeks in a patient with hyperthyroidism.

The cause of ATD-induced agranulocytosis is not fully explained, but an immune-mediated mechanism and direct toxic effects have been proposed. It's reported that an antibody against mature blood cells and bone precursor cells, which targets an antigen on neutrophils, is found in the etiology of the agranulocytosis induced by ATDs [1, 17–19]. Destruction of granulocytes by direct or indirect drug intoxication takes early stage [20]. Most cases of ATD-induced agranulocytosis occur within the first 3 months, but sometimes they can occur the first time after interruption and subsequent resumption of the same ATD treatment [6, 7]. Some studies showed that MMI-induced agranulocytosis is dose-related and the risk is greater in older patients [14, 21]. In a large sample study in the Japan, 754 cases of ATD-induced agranulocytosis developed within 3 months after starting ATD therapy in 84.5% of patients. The mean age was 43.4 years [6]. In the present case, the 51-year-old patient was given total dosage of 840 mg of MMI in 4 weeks and got a fever. This is the most common presenting symptom according to the literature [6, 7, 12–16].

The cause of severe hepatotoxicity in the patient with hyperthyroidism may be multifactorial; it can occur as a reason for hyperthyroidism* per se, *ATD-induced, thyrotoxic heart failure, and other diseases unrelated to hyperthyroidism, such as viral hepatitis, sepsis, alcohol abuse, cholangitis, primary biliary cirrhosis, and autoimmune hepatitis. In this case, the diagnosis complies to the criteria of ATD-induced severe hepatotoxicity defined in the guidelines proposed by Tajiri and Shimizu [8].

The patient had a normal liver function at the beginning of receiving MMI therapy. After taking MMI for 4 weeks, the clinical features indicate that the patient has an ATD-induced severe hepatotoxicity. We also ruled out the other reasons like viral hepatitis infection, alcohol abuse, and so forth. The mechanism of ATD-induced hepatotoxicity is uncertain. Report stated that biotransforming downstream events and their interactions with several environmental and genetic factors could play a role in the proposed model of ATD-induced hepatotoxicity [22]. PTU-related hepatotoxicity takes the form of an allergic hepatitis and it often markedly elevated aminotransferase levels and biopsy specimens show submassive or massive hepatic necrosis. MMI-induced hepatotoxicity is typical of a cholestatic process and is related to dose [10, 23]. The case had a significant abrupt elevation in bilirubin instead of transaminase and slowly recovered after MMI discontinuation, which is in agreement with previous reports [12, 24]. Since it's reported that fatality rate is 23–41% in ATD-induced severe hepatotoxicity, liver transplantation may be required [11, 25, 26]. Colwell et al. [14] reported that a case previous studies of synchronous ATD-induced agranulocytosis and severe hepatotoxicity died due to fulminating septicemia secondary to agranulocytosis and hepatitis. Fortunately, our patient's granulocytes counts, liver function, and thyroid function remain in normal range after cessation of MMI and ablation of the thyroid with ^131^I for the subsequent 6 months. This may be attributed to improved understanding and therapeutic method of MMI-induced severe hepatotoxicity along with agranulocytosis in recent years.

Reports of concurrent agranulocytosis and severe hepatotoxicity in reaction to ATD are scarce. The mechanism is uncertain; according to limited reports, there may be drug-related immune reactions as there are studies which have shown that hypersensitized lymphocytes may produce cholestatic and neutrophil factors on simulation with the antigen [9, 17]. ATD-induced severe hepatotoxicity along with agranulocytosis is potentially lethal because it's acute, its clinical behavior is difficult to be predicted and proceeds rapidly. Patients should be informed of side effects of ATDs, and it has been the standard approach to monitoring of blood cell count and liver function, especially in the first 3 months. If they develop to sore throat, fever, jaundice, dark urine, nausea, and fatigue, they should visit the hospital immediately and be alerted to stop ATD. Our patient was presented to jaundice and the symptoms of sore throat without evidences of other infection. Routine blood cell count and liver function can make a diagnosis.

## 6. Conclusion

This is the case of concurrent occurrence of both agranulocytosis and severe hepatotoxicity induced by MMI therapy after 4 weeks. After being stopped using MMI, changed to be treated with hepatoprotective and antibiotic, ablation of the thyroid with ^131^I, the patient got a favorable prognosis. Physicians need to be aware of the extremely rare, but clinically significant, adverse effects with ATD-induced agranulocytosis and severe cholestatic hepatotoxicity.

## Figures and Tables

**Table 1:** Clinical course of laboratory findings.

Week	Action	FT3	FT4	TSH	ALT	AST	TB	DB	PT	WBC	Granulocytes
Normal range		3.23–7.22 pmol/L	10.3–24.45 pmol/L	0.4–4.0 mIU/L	5–40 U/L	8–40 U/L	0–21 *µ*mol/L	1–5 *µ*mol/L	10–13.5 s	4.0–10.0 × 10^9^/L	2.0–7.0 × 10^9^/L
−4	Diagnosis MMI 20 mg/day started	12.78	56.72	0.001	30	21	12	7	—	4.2	2.2
0	MMI stopped	5.61	37.1	0.001	89	30	385	299	17	1.1	0
1		4.15	30.8	0.009	35	22	390	224	16	2.3	0.5
2		5.86	22.3	0.009	30	22	158	122	—	5.9	3.1
3	^131^I treatment with 8 mCi	9.42	32.8	0.009	37	22	95	42	13	9.1	6.6
5		—	—	—	32	21	20	12	—	5.1	2.9
15		4.4	13.6	0.009	22	23	16	8	—	4.8	2.6
27		4.4	10.6	2.1	18	18	15	7	—	4.9	2.3

FT3: free triiodothyronine; FT4: free thyroxine; TSH: thyroid stimulating hormone; ALT: alanine aminotransferase; AST: aspartate aminotransferase; TB: total bilirubin; DB: direct bilirubin; PT: prothrombin time; WBC: white blood count; “—” means no such examinations were done.

**Table 2:** ATD-induced agranulocytosis and severe hepatotoxicity in patients with hyperthyroidism reported previously in the literature.

	Author					
	Tran et al. [16]	Jain et al. [13]	Vilchez et al. [12]	Colwell et al. [14]	Hoffman et al. [15]	Present case
Age/gender	29/F	45/F	37/F	60/F	55/F	51/M
ATD	CBZ	CBZ	CBZ	PTU	PTU	MMI
Duration of ATD treatment (days)	19	42	15	28	30	28
Daily dose at the time of diagnosis (mg/d)	30	30	30	300	300	20
Laboratory findings at the time of diagnosis						
Granulocyte count (×10^9^/L)	0	0.032	0.08	0.166	0	0
Liver function						
ALT/AST (U/L)	220/97	123/210	44/72	—	165/76	89/30
TB/DB (*µ*mol/L)	104/—	212/133	151/121	186/—	297/—	385/299
Initial treatment						
G-CSF	Yes	No	Yes	—	Yes	No
Antibiotic treatment	Yes	Yes	Yes	—	Yes	Yes
Dose of ^131^I given (MBq)	Yes	No	555	—	444	296
Recovery period to normal from cessation ATD (days)						
Granulocyte count	15	—	34	Death	9	14
Liver function	25	—	69	Death	90	35
Clinical status in thyroid function at follow-up	Subclinical hyperthyroidism	—	Subclinical hyperthyroidism	Death	Euthyroid	Euthyroid

ATD: antithyroid drug; CBZ: carbimazole; PUT: propylthiouracil; MMI: methimazole; ALT: alanine aminotransferase; AST: aspartate aminotransferase; TB: total bilirubin; DB: direct bilirubin; G-CSF: Granulocyte-colony stimulating factor; “—”: not available.

## Abbreviations

ATD: Antithyroid drug
MMI: Methimazole
PTU: Propylthiouracil
CBZ: Carbimazole
FT4: Free thyroxine
FT3: Free triiodothyronine
TSH: Thyroid-stimulating hormone
TB: Total serum bilirubin
DB: Conjugated bilirubin
ALT: Alanine aminotransferase
AST: Aspartate aminotransferase
PT: Prothrombin time
ULN: Upper limit of normal
PT-INR: Prothrombin time international ratio
G-CSF: Granulocyte-colony stimulating factor.
